# Emerging perspectives of synaptic biomarkers in ALS and FTD

**DOI:** 10.3389/fnmol.2023.1279999

**Published:** 2024-01-05

**Authors:** Karrthik Krishnamurthy, Raj Kumar Pradhan

**Affiliations:** Department of Neuroscience, Vickie and Jack Farber Institute for Neuroscience, Thomas Jefferson University, Philadelphia, PA, United States

**Keywords:** amyotrophic lateral sclerosis, frontotemporal dementia, synaptic dysfunction, synaptic biomarkers, TDP-43, neuronal pentraxin, C9orf72, artificial intelligence

## Abstract

Amyotrophic Lateral Sclerosis (ALS) and Frontotemporal Dementia (FTD) are debilitating neurodegenerative diseases with shared pathological features like transactive response DNA-binding protein of 43 kDa (TDP-43) inclusions and genetic mutations. Both diseases involve synaptic dysfunction, contributing to their clinical features. Synaptic biomarkers, representing proteins associated with synaptic function or structure, offer insights into disease mechanisms, progression, and treatment responses. These biomarkers can detect disease early, track its progression, and evaluate therapeutic efficacy. ALS is characterized by elevated neurofilament light chain (NfL) levels in cerebrospinal fluid (CSF) and blood, correlating with disease progression. TDP-43 is another key ALS biomarker, its mislocalization linked to synaptic dysfunction. In FTD, TDP-43 and tau proteins are studied as biomarkers. Synaptic biomarkers like neuronal pentraxins (NPs), including neuronal pentraxin 2 (NPTX2), and neuronal pentraxin receptor (NPTXR), offer insights into FTD pathology and cognitive decline. Advanced technologies, like machine learning (ML) and artificial intelligence (AI), aid biomarker discovery and drug development. Challenges in this research include technological limitations in detection, variability across patients, and translating findings from animal models. ML/AI can accelerate discovery by analyzing complex data and predicting disease outcomes. Synaptic biomarkers offer early disease detection, personalized treatment strategies, and insights into disease mechanisms. While challenges persist, technological advancements and interdisciplinary efforts promise to revolutionize the understanding and management of ALS and FTD. This review will explore the present comprehension of synaptic biomarkers in ALS and FTD and discuss their significance and emphasize the prospects and obstacles.

## Introduction

Amyotrophic Lateral Sclerosis (ALS) and Frontotemporal Dementia (FTD) are two serious neurodegenerative conditions that cause significant suffering and morbidity globally. ALS, also known as Lou Gehrig's disease, primarily affects the neurons responsible for voluntary muscle control, resulting in gradual muscle weakness and eventually respiratory failure (Zarei et al., 2015). In contrast, FTD is a group of disorders that cause a gradual loss of nerve cells in the brain's frontal and temporal lobes, leading to problems with language, behavior, and motor skills (Boxer and Miller, 2005; Wen et al., 2014). While FTD and ALS are distinct disorders, they share some common pathological features, including the presence of transactive response DNA-binding protein of 43 kDa (TDP-43) inclusions and similar genetic mutations (Lillo and Hodges, 2009). In particular, synaptic dysfunction is increasingly recognized as a key element in ALS/FTD pathogenesis (Laszlo et al., 2022; Mora and Allodi, 2023).

There is currently no cure for these diseases and limited treatment options are available, making it urgent to improve our understanding of their underlying mechanisms and identify new therapeutic targets. Biomarkers play a crucial role in providing insights into disease mechanisms, progression, and therapeutic responses. Lately, there has been a focus on a particular type of biomarker known as synaptic biomarkers. These biomarkers consist of proteins related to synaptic function or components of synaptic structure, which shed light on the integrity of synapses (Camporesi et al., 2020). Synapses are essential junctions where neurons communicate, playing a vital role in cognitive and motor functions (Batool et al., 2019). Any alterations that occur at the synaptic level can indicate the beginning and advancement of neurodegenerative diseases.

The exploration of synaptic biomarkers in ALS and FTD brings about both possibilities and obstacles. These biomarkers have the potential to detect the disease early, monitor its progression, and evaluate therapeutic responses. However, studying these biomarkers is not without challenges. From the limitations in detecting and measuring them to the difficulty of translating findings from animal models to humans, there are significant hurdles to overcome.

## Synaptic dysfunction as a converging mechanism in ALS/FTD

Synaptic dysfunction is increasingly recognized as a converging mechanism in various genetic forms of ALS and FTD (Ling et al., 2013; Krishnamurthy et al., 2021; Gelon et al., 2022). Several genes linked to familial forms of ALS and FTD encode proteins that have roles at the synapse or are associated with synaptic function (Sephton and Yu, 2015; Gan et al., 2018; Casci et al., 2019; Ionescu et al., 2023).

### TDP-43

The TDP-43 protein has also been implicated in synaptic function (Ling, 2018; Strah et al., 2020; Broadhead et al., 2022). Abnormal aggregation of TDP-43 in the cytoplasm of neurons is a key pathological feature of most ALS cases and a subset of FTD cases (Mackenzie and Rademakers, 2008). In a study by Feiler et al. (2015), TDP-43 was shown to undergo synaptic intercellular transmission and prion-like seeding.

### Fused in sarcoma

FUS is another RNA-binding protein associated with familial forms of ALS and FTD (Da Cruz and Cleveland, 2011; Daigle et al., 2016). It has been implicated in the regulation of synaptic activity and local protein translation at synapses (Sephton et al., 2014). Super-resolution imaging and CLIP-seq revealed that FUS localizes near the vesicle reserve pool at synapses, influencing synapse organization and plasticity. Increased synaptic FUS during early ALS disease in a mouse model led to altered GABAergic synapses, indicating that synaptic FUS accumulation may trigger neurodegeneration (Sahadevan et al., 2021).

### C9orf72

The most common genetic cause of both familial ALS and FTD is a hexanucleotide repeat expansion in the *C9orf72* gene (Balendra and Isaacs, 2018). Recently, in a zebrafish model with reduced C9orf72 function, synaptic vesicle trafficking and release at the neuromuscular junction were affected, shedding light on its role in synaptic function (Butti et al., 2021). Evidence from studies on C9orf72 models indicates pathological changes, including altered dendritic spines, enhanced excitotoxicity, and increased activity of extrasynaptic NMDA receptors, suggesting that synaptic disturbances may be involved in FTD (Huber et al., 2022).

### Tau

While more commonly associated with Alzheimer's disease, mutations in the *MAPT* gene, which encodes the Tau protein, have been linked to some cases of FTD (Mackenzie and Neumann, 2016). Tau is known to regulate synaptic function and plasticity (Brunello et al., 2020; Robbins et al., 2021) and Tau pathology is associated with synaptic loss (Coomans et al., 2021; Wu et al., 2021).

In summary, multiple lines of evidence suggest that synaptic dysfunction may be a converging mechanism in various genetic forms of ALS and FTD. However, further research is needed to fully elucidate the roles of these and other synaptic proteins in disease pathogenesis and to understand how synaptic dysfunction contributes to the clinical features of these diseases.

## Overview of synaptic biomarkers

Synaptic biomarkers represent quantifiable indicators of synaptic function or structure (Colom-Cadena et al., 2020). Synapses are critical for neurotransmission and are essential for the cognitive and motor functions of the CNS. Proteins involved in the transmission of signals across synapses, as well as those forming the synaptic architecture, can serve as synaptic biomarkers. Alterations in the concentration or activity of these biomarkers can offer valuable information on the functional integrity of synapses and the overall neuronal network (Camporesi et al., 2020).

The significance of synaptic biomarkers in understanding disease pathogenesis is multi-fold. First, synaptic dysfunction is often an early feature in many neurodegenerative diseases, preceding neuronal loss. Therefore, synaptic biomarkers can potentially provide early indicators of disease onset (Galasko et al., 2019). Second, they can shed light on the mechanisms of synaptic damage in disease conditions, offering a deeper understanding of disease pathogenesis (Taoufik et al., 2018). Finally, the correlation of synaptic biomarkers with disease progression can help to map the trajectory of the disease, contributing to better prognostic models. Synaptic biomarkers can play a critical role in the development of therapeutics for ALS and FTD (Krishnamurthy and Pasinelli, 2021; Das et al., 2023).

## Methods for studying synaptic biomarkers in ALS/FTD

Our understanding of synaptic dysfunction in ALS is mainly based on post-mortem immunohistochemistry (Aousji et al., 2023) but this approach cannot be applied in living subjects. Synaptic biomarkers are studied in ALS either through non-invasive or invasive methods. Broadly these methods can be classified as imaging-based or fluid-based. Through these methods we can gain valuable insights into synaptic changes in living individuals with ALS/FTD and track disease progression over time (Sturmey and Malaspina, 2022; Mead et al., 2023). Table 1 presents an overview of various methods to study synaptic biomarkers in ALS/FTD, along with their advantages and limitations.

**Table 1:** Overview of methods to study synaptic biomarkers in ALS/FTD.

**Method**	**Advantages**	**Limitations**
Immunohistochemistry	Provides direct visualization of protein expression and distribution in brain tissue. Allows examination of protein aggregates and inclusions characteristic of ALS/FTD.	Invasive and requires post-mortem brain tissue. Limited to studying fixed tissue, not dynamic changes.
Enzyme-linked immunosorbent assay (ELISA)	Quantitative measurement of protein levels in cerebrospinal fluid (CSF) or blood. Non-invasive and easily accessible sample collection.	May not reflect synaptic-specific changes and can be influenced by overall protein levels in the sample. Limited spatial resolution and cannot distinguish changes in specific brain regions.
Mass spectrometry (MS)	Comprehensive profiling of proteins in biological samples. Can identify novel proteins or changes in protein expression that may be relevant to ALS/FTD.	Requires sophisticated equipment and expertise in data analysis. May not provide information on the functional state of proteins.
Single-molecule array (SIMOA)	Extremely sensitive and can detect low concentrations of proteins in CSF or blood. Potential for early detection of disease-specific changes in synaptic biomarkers.	Requires specialized equipment and expertise in data analysis. Limited to known proteins and may not identify novel markers.
Positron emission tomography (PET)	Provides insights into related pathological processes or protein accumulations in the brain. Non-invasive imaging technique with the ability to study brain regions affected by ALS/FTD.	Requires specialized radiotracers and may not directly visualize specific synaptic biomarkers. PET tracers for disease-specific synaptic biomarkers are still under development.
Neuroimaging (a) Functional magnetic resonance imaging (fMRI) (b) Diffusion Tensor Imaging (DTI)	fMRI provides information on brain activity and functional connectivity related to synaptic function. DTI measures white matter integrity, which is critical for synaptic communication.	Cannot directly measure synaptic proteins and offers indirect information on synaptic dysfunction. Changes in brain activity and connectivity may not specifically indicate synaptic dysfunction.
Bioinformatics	Allows integration and analysis of complex datasets, including genomic, proteomic, and imaging data. Can identify potential associations and correlations between synaptic biomarkers and disease progression.	Requires specialized computational skills and may involve complex data processing. Results need to be validated using independent datasets and experimental approaches.

### Imaging-based biomarker analysis

Neuroimaging techniques either report a specific biomarker or can reveal the overall status of synaptic structure and function in living subjects (Young et al., 2020). For example, Magnetic Resonance Imaging (MRI) can reveal changes in brain anatomy associated with synaptic loss or degeneration in ALS/FTD (Trojsi et al., 2012; Rajagopalan et al., 2013). Functional magnetic resonance imaging (fMRI) on the other hand can assess functional connectivity between brain regions related to synaptic activity (Peet et al., 2021). Diffusion Tensor Imaging (DTI) can investigate the integrity of white matter tracts connecting brain regions involved in synaptic transmission (Alruwaili et al., 2019). Positron Emission Tomography (PET) uses specific radiotracers to measure synaptic density or the presence of abnormal protein aggregates such as tau (Ni and Nitsch, 2022). PET imaging using radiotracers that bind to synaptic markers, such as synaptic vesicle glycoprotein 2A (SV2A) or synaptic density markers, can help visualize and quantify synaptic density and alterations in ALS/FTD (Serrano et al., 2022).

### Fluid-based biomarker analysis

Fluid based biomarker analysis involves the use of bodily fluids such as blood and cerebrospinal fluid (CSF) that can be used to study levels of certain synaptic proteins or biomarkers that may correlate with synaptic dysfunction. Lumbar puncture allows the collection of CSF, which can be analyzed for synaptic biomarkers, neurotransmitters, or protein aggregates (Camporesi et al., 2020). Enzyme-Linked Immunosorbent Assay (ELISA) is one of the most commonly used technique used to study biomarkers associated with ALS and FTD allowing for the sensitive and quantitative measurement of specific proteins or molecules in biological samples. Magnetic Resonance Spectroscopy (MRS) allows researchers to measure the levels of various metabolites, including neurotransmitters like glutamate and GABA, which are essential for synaptic function (Pasanta et al., 2023). Another method of significant potential is targeted mass spectrometry (MS) which was recently utilized to explore 15 candidate synaptic biomarkers in CSF from patients with Parkinson's disease, corticobasal degeneration, progressive supranuclear palsy, multiple system atrophy, and Alzheimer's disease (Nilsson et al., 2023).

## Synaptic biomarkers in ALS

Numerous studies have been carried out to discover synaptic biomarkers that could be indicative of ALS. A growing body of studies suggests elevated levels of neurofilament light chain (NfL) in the CSF and blood of ALS patients. The levels of NfL were found to correlate with the progression of the disease, indicating that it could be a valuable biomarker for ALS (Sun et al., 2020; Dreger et al., 2021; Zhou et al., 2021; Thompson et al., 2022). The elevation of NfL is believed to reflect axonal damage resulting from motor neuron degeneration (Verde et al., 2021). Furthermore, TDP-43 has emerged as another important biomarker in ALS (Kasai et al., 2009). TDP-43 forms pathological inclusions in neurons in most ALS cases and its mislocalization may be associated with synaptic dysfunction (Lépine et al., 2023).

Research has shown the usefulness of synaptic biomarkers in understanding the progression and severity of ALS. For instance, individuals with higher NfL levels at diagnosis experienced a faster rate of disease progression (Lu et al., 2015; Sugimoto et al., 2020). This highlights the potential of this biomarker in monitoring disease progression and predicting prognosis. Additionally, evidence suggests that the amount of TDP-43 pathology is linked to the clinical symptoms of the disease, making it a promising biomarker for determining disease severity.

NfL and TDP-43 are two examples of biomarkers that are present in both ALS and other disorders like FTD (Katzeff et al., 2022). However, the particular characteristics of these markers, such as the pattern of TDP-43 pathology, suggest that they may be unique to ALS. Moreover, there are newly discovered potential synaptic biomarkers that could be specific to ALS, such as vesicle-associated membrane-protein-associated protein B (VAPB) which was absent in Parkinson's disease patient peripheral blood mononuclear cells (Cadoni et al., 2020). In a recent study, researchers used unbiased discovery-based methods along with targeted quantitative comparative analyses on CSF samples from ALS patients and healthy control individuals leading to the identification of 53 differential proteins between the two groups after CSF fractionation. These proteins included both previously known ones, validating the approach, and novel proteins with potential as new biomarkers (Oh et al., 2023). Next, the researchers used parallel reaction monitoring (PRM) mass spectrometry to further examine the identified proteins. Fifteen proteins were found to show significant differences between ALS and control groups. These proteins are: APOB, APP, CAMK2A, CHI3L1, CHIT1, CLSTN3, ERAP2, FSTL4, GPNMB, JCHAIN, L1CAM, NPTX2, SERPINA1, SERPINA3, and UCHL1. Among these CLSTN3, NPTX2, CAMK2A have synaptic roles. Further investigation is necessary to verify these biomarkers and comprehend their precise functions in ALS pathology.

## Synaptic biomarkers in frontotemporal dementia

As with ALS, several studies have aimed to identify potential synaptic biomarkers in FTD. Of these, TDP-43 has been the most studied, given its presence in neuronal inclusions in the majority of FTD cases (Neumann et al., 2006). Mislocalization and accumulation of TDP-43 in neurons are thought to disrupt synaptic function, potentially contributing to the cognitive and behavioral symptoms observed in FTD. Additionally, tau proteins, particularly in their hyperphosphorylated form, have been implicated in certain subtypes of FTD, known as tauopathies (Schraen-Maschke et al., 2008). The balance between phosphorylated tau (p-tau) and total tau (t-tau) in the CSF has been suggested as a potential biomarker for these cases (Hampel et al., 2010). Recent research has attempted to correlate synaptic biomarker levels with disease progression and severity in FTD. Interestingly, one study found that changes in CSF tau levels correlated with the progression of symptoms in tau-positive FTD, suggesting its potential role as a disease progression marker (Katzeff et al., 2022). More research is needed to understand how these and other potential synaptic biomarkers may correlate with disease progression and severity in FTD.

While FTD shares several potential synaptic biomarkers with ALS, such as TDP-43 and NfL, others are more unique to FTD. For example, tau proteins, especially in their hyperphosphorylated form, are particularly relevant to certain subtypes of FTD but not ALS (Bodea et al., 2016). Understanding these unique and common synaptic biomarkers can improve our understanding of these diseases, provide diagnostic and prognostic tools, and possibly highlight new therapeutic targets.

Neuronal pentraxins (NPs) are a family of proteins that play a crucial role in synaptic remodeling and homeostasis. They are named after the complement proteins due to their similar structure (Zhou et al., 2023). The family includes neuronal pentraxin 1 (NP1), neuronal pentraxin 2 (NPTX2), and neuronal pentraxin receptor (NPTXR). They have been shown to cluster at excitatory synapses and play a role in synaptic plasticity (Gómez de San José et al., 2022).

Lower levels of NPTX2 have been reported in the CSF of patients with FTD, suggesting a potential role as a synaptic biomarker (van der Ende et al., 2020). NPTXR, an integral component of the NP complex, is expressed primarily in neurons. Its expression level has been shown to correlate with cognitive function in FTD. Changes in NPTXR expression could therefore potentially serve as a marker of synaptic integrity in diseases like FTD (Sogorb-Esteve et al., 2022). In a recent study, Single-cell transcriptomics revealed a set of misregulated RNA targets shared by TDP-43 overexpressing neurons and patient brains with TDP-43 pathology, with the synaptic protein NPTX2 being consistently misaccumulated in ALS and FTD patient neurons providing a direct link between TDP-43 misregulation and NPTX2 accumulation (Hruska-Plochan et al., 2021).

## Opportunities for synaptic biomarkers in ALS and FTD

One of the significant opportunities presented by synaptic biomarkers lies in their potential for early detection of ALS and FTD. Given that synaptic dysfunction often precedes neuronal loss, the presence of altered synaptic biomarkers could signal disease onset before the appearance of clinical symptoms. Additionally, biomarkers like NfL that correlate with disease progression could provide valuable prognostic information, guiding patient care and management (Swift et al., 2021; Vignaroli et al., 2023).

Synaptic biomarkers could pave the way for personalized medicine in ALS and FTD. By understanding how specific biomarkers correlate with disease progression and severity, treatments could be tailored based on a patient's unique biomarker profile (Das et al., 2023). This individualized approach could potentially lead to improved outcomes, mitigating disease progression and enhancing the quality of life for patients.

The study of synaptic biomarkers can also significantly contribute to drug development and clinical trials. By serving as objective measures of disease state or response to therapy, these biomarkers can provide critical insights into a drug's mechanism of action or efficacy (Colom-Cadena et al., 2020). For instance, reductions in biomarker levels following drug administration could indicate therapeutic effect, while no change might suggest a lack of efficacy. In this way, synaptic biomarkers can play a crucial role in the design and evaluation of clinical trials.

Perhaps one of the most exciting opportunities provided by synaptic biomarkers is the chance to further unravel the complex disease mechanisms underlying ALS and FTD. By exploring how these biomarkers change in response to disease progression and how they relate to one another, scientists can build a more complete picture of the molecular and cellular changes that drive these neurodegenerative diseases. This deeper understanding could, in turn, drive the development of new therapeutic strategies aimed at mitigating disease progression or even preventing disease onset. Table 2 provides a summary of the utility of synaptic biomarkers in therapeutic Development.

**Table 2:** Summary of the utility of synaptic biomarkers in therapeutic development.

**Utility of synaptic biomarkers in therapeutic development**	**Examples**	**Limitations**
Disease understanding	TDP-43 pathology in ALS and FTD. NfL as a marker of axonal damage. Tau proteins in tauopathies.	Complexity of disease pathogenesis makes it challenging to establish causality between biomarkers and disease. Biomarkers may be influenced by confounding factors such as age, gender, and comorbidities. Biomarker levels may vary at different stages of the disease, requiring longitudinal studies.
Disease progression and severity	NfL levels correlating with ALS progression. TDP-43 pathology correlating with clinical symptoms in ALS. Changes in tau levels reflect FTD progression.	Variability in biomarker levels across individuals and disease stages makes it challenging to define universal reference values or cut-offs. Biomarker levels may be influenced by factors unrelated to disease progression, affecting interpretation. Limited availability of validated biomarker assays may hinder widespread use.
Drug targets	Identifying TDP-43 as a target for ALS therapeutics. Targeting synaptic proteins to improve synaptic function. Potential for drug development aimed at modulating synaptic biomarkers.	Not all synaptic biomarkers are directly modifiable drug targets, limiting their immediate application. Modulating synaptic biomarkers may have unintended consequences on other synaptic processes.
Patient stratification	Identifying subgroups of ALS or FTD patients based on unique synaptic biomarker profiles. Enabling more targeted therapeutic approaches.	Biomarkers may not be specific to certain subgroups and may not accurately stratify patients. Small sample sizes in some studies can limit generalizability. Replication studies are essential to confirm the utility of synaptic biomarkers for patient stratification.
Treatment response	Biomarker changes indicating response to therapy in clinical trials.	Biomarkers may respond differently to various therapeutic approaches, requiring careful interpretation. Some synaptic biomarkers may not show immediate changes following treatment, necessitating long-term monitoring.
Early detection	NfL as a potential early diagnostic marker for ALS. Early changes in synaptic biomarkers in FTD subtypes. Enabling early therapeutic Intervention.	Biomarker levels may not be significantly altered in the very early stages of the disease. Biomarkers' diagnostic accuracy may vary in different patient populations or disease subtypes.
Personalized Medicine	Tailoring treatment based on individual biomarker profiles. Stratifying patients based on unique synaptic biomarkers. Improving treatment outcomes.	Biomarker profiles may not completely encompass the heterogeneity of ALS and FTD. Identifying the most informative biomarkers for personalized treatment may be challenging.
Clinical trial endpoints	Using synaptic biomarkers as surrogate endpoints in clinical trials. Accelerating drug development process.	Establishing the validity of synaptic biomarkers as reliable clinical trial endpoints may require extensive validation and regulatory approval.

## Challenges in synaptic biomarker research in ALS and FTD

One of the primary challenges in synaptic biomarker research is the technological limitation in detection and measurement. Synaptic biomarkers often require sensitive and specific assays for accurate detection. However, these biomarkers are usually present in minute quantities, and their levels may vary based on various factors, including the time of sample collection, sample handling, and patient-specific factors. This necessitates the development of more sensitive and robust assays for their detection (Klyucherev et al., 2022).

There is considerable variability in biomarker levels across different patients and stages of diseases, adding to the complexity of interpreting these markers. Factors such as genetic heterogeneity, disease subtype, age, gender, and comorbidities can influence biomarker levels, making it challenging to establish universally applicable cut-offs or reference values. Moreover, understanding how biomarker levels change over the course of the disease and correlating these changes with clinical progression is a complex task (Mayeux, 2004; McDermott et al., 2013).

While animal models have greatly contributed to our understanding of ALS and FTD, translating these findings to humans has been challenging. The pathophysiology of these diseases in animal models may not perfectly mimic the human condition, and hence, the biomarkers identified in these models may not be relevant in humans. Thus, findings from animal studies must be interpreted cautiously and validated in human populations (Bonifacino et al., 2021).

Many biomarker studies are based on small sample sizes, and their findings may not be generalizable to larger, more diverse patient populations. Therefore, there is a pressing need for larger, multi-center studies to validate these markers. Additionally, replication studies are crucial to ensure the reliability of these findings (Freidlin et al., 2010). As biomarker research is a rapidly evolving field, continuous re-evaluation and updating of these markers based on the latest evidence is necessary.

## Machine learning and artificial intelligence approaches in synaptic biomarker discovery

ML and AI have the potential to significantly accelerate synaptic biomarker discovery and therapeutic development for ALS and FTD. These advanced computational approaches can process large datasets and identify patterns and relationships that may be difficult to uncover with traditional methods.

For example, ML/AI algorithms can analyze complex multi-omics data, including genomic, proteomic, and neuroimaging data, to find novel synaptic biomarkers associated with ALS/FTD (Grollemund et al., 2019). By integrating diverse datasets, ML/AI models can reveal previously unknown connections between genes, proteins, and disease features, as has been shown in cancer research (Arjmand et al., 2022).

ML/AI techniques can also analyze clinical, imaging, and biomarker data to predict disease progression and estimate the likelihood of developing ALS/FTD (Rajagopalan et al., 2023). Early and accurate diagnosis and prognosis can inform personalized treatment strategies to improve patient outcomes.

Additionally, ML/AI can efficiently screen chemical libraries to identify potential ALS/FTD drug candidates and repurpose existing medications that may have therapeutic effects (Vatansever et al., 2021). By integrating molecular and genetic data, ML/AI can predict the most promising targets involved in synaptic dysfunction to prioritize for drug development. ML/AI can also optimize clinical trial design, including patient selection and treatment dosing, to increase the probability of success (Davenport and Kalakota, 2019). Analyzing patient-specific data with AI algorithms allows for personalized medicine by predicting individual responses to treatments.

Finally, ML/AI can quantify synaptic density and structural changes in the brain from neuroimaging data to track disease progression and monitor treatment efficacy (Boyle et al., 2021; Monsour et al., 2022). In summary, ML/AI has diverse applications that can accelerate nearly every aspect of ALS/FTD research and drug development, from biomarker discovery to personalized therapeutics.

## Future directions

Emerging technologies offer new avenues for improving the detection and measurement of synaptic biomarkers. Advances in genomics, proteomics, and metabolomics, coupled with powerful bioinformatics tools, are enabling the identification and validation of biomarkers at an unprecedented scale and speed. Additionally, the development of more sensitive and specific immunoassays, such as single-molecule array (SIMOA) technology, can greatly enhance our ability to detect minute quantities of these biomarkers (Wilson et al., 2016; Wu et al., 2022). Continued technological innovation will undoubtedly play a crucial role in the future of synaptic biomarker research.

Looking forward, there are several exciting new areas of synaptic biomarker research. For instance, exploring the interplay between synaptic biomarkers and other pathophysiological processes, such as inflammation or oxidative stress, could provide novel insights into disease mechanisms. Additionally, the emerging field of neuroimaging biomarkers could complement biochemical biomarker studies, providing a more holistic picture of disease progression. Lastly, research into biomarkers of synaptic resilience or compensatory mechanisms could open new avenues for therapeutic intervention.

Addressing the current challenges in synaptic biomarker research could significantly shape the future direction of this field. For example, overcoming technological limitations could allow for the detection of previously unidentified biomarkers or more accurate quantification of known ones. Tackling variability issues could lead to the development of personalized biomarker profiles, providing more precise diagnostic and prognostic information. Successfully translating findings from animal models to humans could accelerate the clinical application of these biomarkers, and large-scale validation studies could solidify their place in clinical practice. Thus, the future of synaptic biomarker research is contingent upon overcoming the present challenges, offering a promising and exciting path forward.

## Author contributions

KK: Conceptualization, Resources, Visualization, Writing—original draft, Writing—review & editing. RP: Resources, Visualization, Software, Writing—review & editing.
